# Recruitment of the rhizo-microbiome army: assembly determinants and engineering of the rhizosphere microbiome as a key to unlocking plant potential

**DOI:** 10.3389/fmicb.2023.1163832

**Published:** 2023-05-05

**Authors:** Inmyoung Park, Young-Su Seo, Mohamed Mannaa

**Affiliations:** ^1^School of Food and Culinary Arts, Youngsan University, Busan, Republic of Korea; ^2^Department of Integrated Biological Science, Pusan National University, Busan, Republic of Korea; ^3^Department of Plant Pathology, Faculty of Agriculture, Cairo University, Giza, Egypt

**Keywords:** rhizosphere microbiome, rhizo-microbiome transplantation, root exudates, rhizosphere microbiome engineering, rhizo-microbiome recruitment

## Abstract

The viable community of microorganisms in the rhizosphere significantly impacts the physiological development and vitality of plants. The assembly and functional capacity of the rhizosphere microbiome are greatly influenced by various factors within the rhizosphere. The primary factors are the host plant genotype, developmental stage and status, soil properties, and resident microbiota. These factors drive the composition, dynamics, and activity of the rhizosphere microbiome. This review addresses the intricate interplay between these factors and how it facilitates the recruitment of specific microbes by the host plant to support plant growth and resilience under stress. This review also explores current methods for engineering and manipulating the rhizosphere microbiome, including host plant-mediated manipulation, soil-related methods, and microbe-mediated methods. Advanced techniques to harness the plant's ability to recruit useful microbes and the promising use of rhizo-microbiome transplantation are highlighted. The goal of this review is to provide valuable insights into the current knowledge, which will facilitate the development of cutting-edge strategies for manipulating the rhizosphere microbiome for enhanced plant growth and stress tolerance. The article also indicates promising avenues for future research in this field.

## 1. Introduction

The rhizosphere is the microenvironment that is intricately shaped by the plant, its surrounding soil, and biotic and abiotic factors. The rhizosphere is one of the most active and ever-changing environments on Earth (Qu et al., 2020). The soil is a rich reservoir of microbial diversity. Each teaspoon of soil contains approximately 1 × 10^9^ microorganisms, similar to the human population of Africa (Nature Reviews Microbiology, 2011). This fact highlights the vast abundance and ecological significance of microbial life in the soil, as well as their potential in various applications. Many microorganisms in the rhizosphere have a neutral impact on the plant, serving as key components of the intricate food web sustained by root exudates. The rhizosphere also harbors both pathogenic and beneficial microorganisms, which significantly influence plant growth and overall health (Philippot et al., 2013). Pathogenic organisms, such as fungi, oomycetes, bacteria, and nematodes, can negatively impact plant growth and health. Beneficial microorganisms, such as nitrogen-fixing bacteria, endo- and ectomycorrhizal fungi, and plant growth-promoting rhizobacteria and fungi, can enhance plant growth and health (Raaijmakers et al., 2009). The abundance and diversity of these microorganisms are dependent on the quantity and quality of rhizodeposits, as well as the outcome of complex interactions within the rhizosphere (Bais et al., 2006).

Given the critical role that the rhizosphere plays in shaping plant growth and health, scientists from a wide range of disciplines have sought to understand the underlying processes that determine the composition, dynamics, and activity of the rhizosphere microbiome. Assembly of the rhizosphere microbiome is orchestrated by many factors, including the genetic makeup of the host plant, the chemical and physical properties of the soil, and the diversity and composition of the resident and bulk soil microorganisms (Haichar et al., 2008; Inceoglu et al., 2012). These factors not only control the assembly process of the rhizosphere microbiome but also have a significant impact on its functional capacity (Yan et al., 2017). A thorough understanding of these factors and how they interact with one another provides valuable insights that could be utilized in developing advanced strategies to manipulate the rhizosphere microbiome for enhanced plant growth and stress tolerance. This is a promising avenue of research.

The assembly and function of the rhizosphere microbiome are profoundly influenced by various factors in the rhizosphere, including host plant, soil, and microbial variables. Methods for manipulating and modulating the rhizosphere microbiome also focus on these key drivers (Bano et al., 2021). Host plant-mediated manipulation demands an understanding of the significant impact of the host plant on shaping the rhizosphere microbiome. This goal has led to the development of techniques for manipulating host plants to recruit beneficial microbiomes (Dubey and Sharma, 2021). These techniques include the use of external elicitors to alter plant physiology, plant breeding methods that incorporate the selection of advantageous rhizosphere microbiomes as a trait, and technological advancements that include genetic engineering tools aimed at enhancing the plant recruitment of useful microbes (Badri et al., 2009a; Geddes et al., 2019; Mannaa et al., 2020). The recent emergence of advanced gene editing tools, such as clustered regularly interspaced short palindromic repeats (CRISPR), has created opportunities for more precise applications to improve plant characteristics, including the recruitment of beneficial rhizosphere microbiomes (El-Mounadi et al., 2020; Zhong et al., 2022).

Concerning soil-related methods, manipulation of the rhizosphere soil to exploit the benefits to plants is an ancient practice that predates the knowledge and understanding of the microbiome. Historical agricultural practices have altered the soil properties to augment plant productivity (Luo et al., 2018). These practices include soil amendments, which have evolved from traditional organic amendments, to more advanced nano-compounds that have the potential to enhance rhizosphere and microbial activity (Rajput et al., 2021).

With respect to microbe-mediated methods, the concept of suppressive soils and the association of the suppression of plant pathogens by microbial agents in the soil have led to the implementation of methods to leverage benefits from these beneficial organisms (Gómez Expósito et al., 2017). This has resulted in numerous efforts to isolate, screen, and characterize microbes from soil and the rhizosphere to improve plant growth and health (Bulgarelli et al., 2015). These efforts have identified numerous biocontrol microbial agents. These agents have demonstrated considerable success, although their success in field application has been limited. The advancement of molecular tools, metagenomic approaches, and a deeper understanding of the entire community has led to the proposal of novel methods, such as mixed consortia inoculation and the innovative approach of rhizo-microbiome transplantation (Jiang et al., 2022).

As scientific understanding and technological capabilities continue to evolve, the deliberate manipulation of the rhizosphere is expected to become increasingly sophisticated. A holistic and systemic approach that considers the interrelated dynamics between plants, soils, and microorganisms is necessary for optimal results. This approach requires an understanding of the complex interactions between host plants, soil, and microbiome to design and implement interventions that promote growth, adaptation, and resilience under harsh conditions or tolerance to stress and diseases (Kumar and Dubey, 2020). With the increasing need for food security and the challenging global environment, rhizosphere engineering is becoming an attractive field of research with the potential to contribute to improved crop quality and productivity.

This review aims to provide a comprehensive overview of the current understanding of the assembly and function of the rhizosphere microbiome and to highlight key factors that influence rhizosphere composition, dynamics, and activity. Additionally, this review discusses various methods for manipulating and modulating the rhizosphere microbiome, including host plant-mediated manipulation, soil-related methods, and microbe-mediated methods. The review also explores the impact of recent advancements in technology, including gene editing and rhizo-microbiome transplantation approaches, on the manipulation of the rhizosphere microbiome to enhance plant growth and stress tolerance. The ultimate goal of this review is to provide valuable insights that can be utilized in the development of advanced strategies for manipulating the rhizosphere microbiome and to highlight productive areas of future research.

## 2. Assembly of rhizosphere microbiome composition and functional capacity

**Figure 1:**
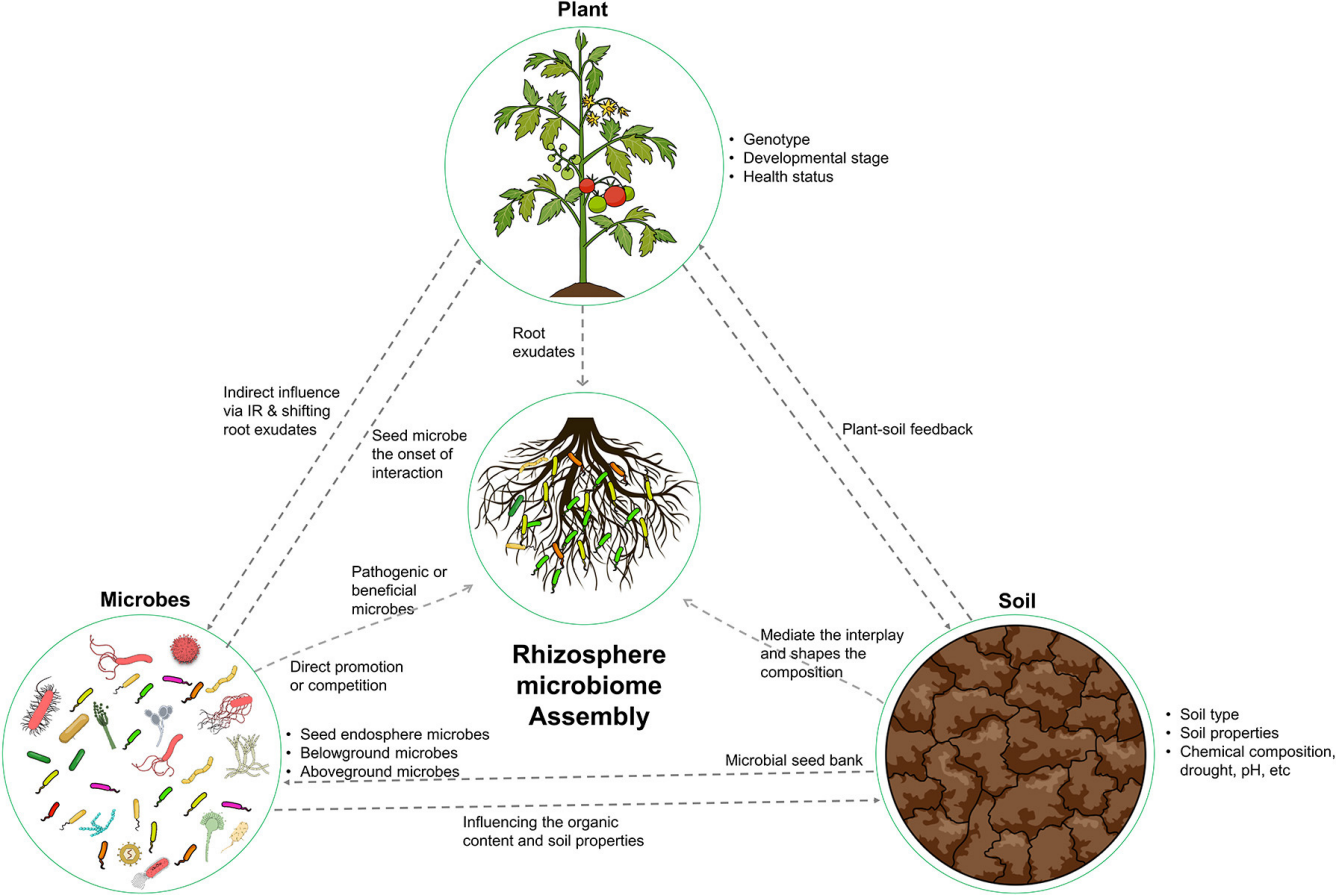
Illustration of the key factors that shape the composition and functional capacity of the rhizosphere microbiome. The host plant, soil, and microbes all play important roles in shaping this ecosystem. The host plant provides nutrients and compounds for microbial recruitment. The soil provides a physical and chemical environment. The microbes interact with both the plant and soil. These determinants work together to create a dynamic and complex ecosystem.

The ecological concept of “everything is everywhere, the environment selects” was proposed by Beijerinck in 1913. This concept highlights the ubiquitous distribution of microorganisms and the role of environmental factors in shaping microbial biogeography (Fierer and Jackson, 2006). This principle is particularly relevant in the context of the rhizosphere microbiome, where the assembly of microorganisms is intricately linked to the surrounding ecological variables, including the host plant, soil, and resident microorganisms (Figure 1). To fully comprehend the complexity of the rhizosphere microbiome, it is necessary to consider the diversity and abundance of microorganisms present and the influence of the plant, soil, and other microorganisms on their distribution and function.

### 2.1. Roles of the host plant

#### 2.1.1. Root exudates

The main factor to differentiate the rhizosphere from bulk soils is the presence of the plant roots. Through rhizodeposition and enrichment with energy and carbon sources, plant roots are primarily responsible for the assembly of the rhizosphere microbial composition (Bais et al., 2006). Unlike bulk soils, the rhizosphere is enriched with copiotrophic microbes, representing the main functional groups within the rhizosphere (Ling et al., 2022). In contrast, the bulk soil generally encompasses a relatively more diverse microbial community with oligotrophic microbes affected mainly by their limited carbon microenvironment independent of the influence of plant roots (Richter et al., 2018). The bulk soil represents the reservoir of microbes that are used to establish the rhizosphere microbiota by the selective activity of the root exudates and the ecological conditions (Mendes et al., 2014; Hamonts et al., 2018). The distinction in the ecological conditions between bulk soils and the rhizosphere could explain the greater functional capacity of the microbial communities of the rhizosphere. The higher release of readily available energy and carbon in different forms, including amino acids, carbohydrates, and sugars, fuels the beneficial microbes in the rhizosphere. These, in turn, perform crucial tasks, such as nitrogen fixation and nutrient solubilization (Nuccio et al., 2020). Alternatively, the presence of plant root debris and associated lignin, cellulose, and hemicellulose within the rhizosphere represents the substrate and selection factor of microbial species with the enzymatic potential to degrade such compounds, including phytopathogens (Ling et al., 2022). Thus, it is important to consider both aspects of the micro-environmental conditions in the rhizosphere, which mainly facilitate beneficial microbes, but also influence phytopathogens.

The root exudates can be considered the tools and messengers by which plants regulate and orchestrate their interactions with the surrounding environment, other plants, and soil microbes. The exudates act as repellents or attractants of specific microbes to shape the rhizo-microbiome composition. Exudates also mediate the linkage with other biological molecules that perform various biological processes that benefit the plant, such as nutrition and defense (Olanrewaju et al., 2019). The composition and extent of root exudates depend on several plant-related factors, including genotype, developmental stage, health status, and mode of photosynthesis (Feng et al., 2021). Moreover, the secretion of root exudates, especially low molecular weight defense compounds, is tightly regulated in plants and involves a complex process of stimuli-based alternations as a strategy for saving energy. An example is the secretion of certain antimicrobials upon pathogen attack (Baetz and Martinoia, 2014). Defense phytochemicals are synthesized in response to a trigger by pathogens; an example is phytoalexins, such as phenylpropanoids. Other defense compounds (i.e., phytoanticipins, such as the diterpene rhizathalene A) are continuously secreted in the root system, even in the absence of the pathogen. These compounds represent the constitutive direct defense of the root system. The absence of the constitutive compounds could indicate higher plant susceptibility to certain pathogens (VanEtten et al., 1994; Badri et al., 2009b; Vaughan et al., 2013). Pathogen infection can also trigger higher production of certain constitutive phytoanticipins; an example is the increased production of momilactone A by rice plants. These constitutive compounds play a role in the suppression of pathogens and provide a competitive advantage for root establishment (Kato-Noguchi et al., 2008; Hasegawa et al., 2010).

**Figure 2:**
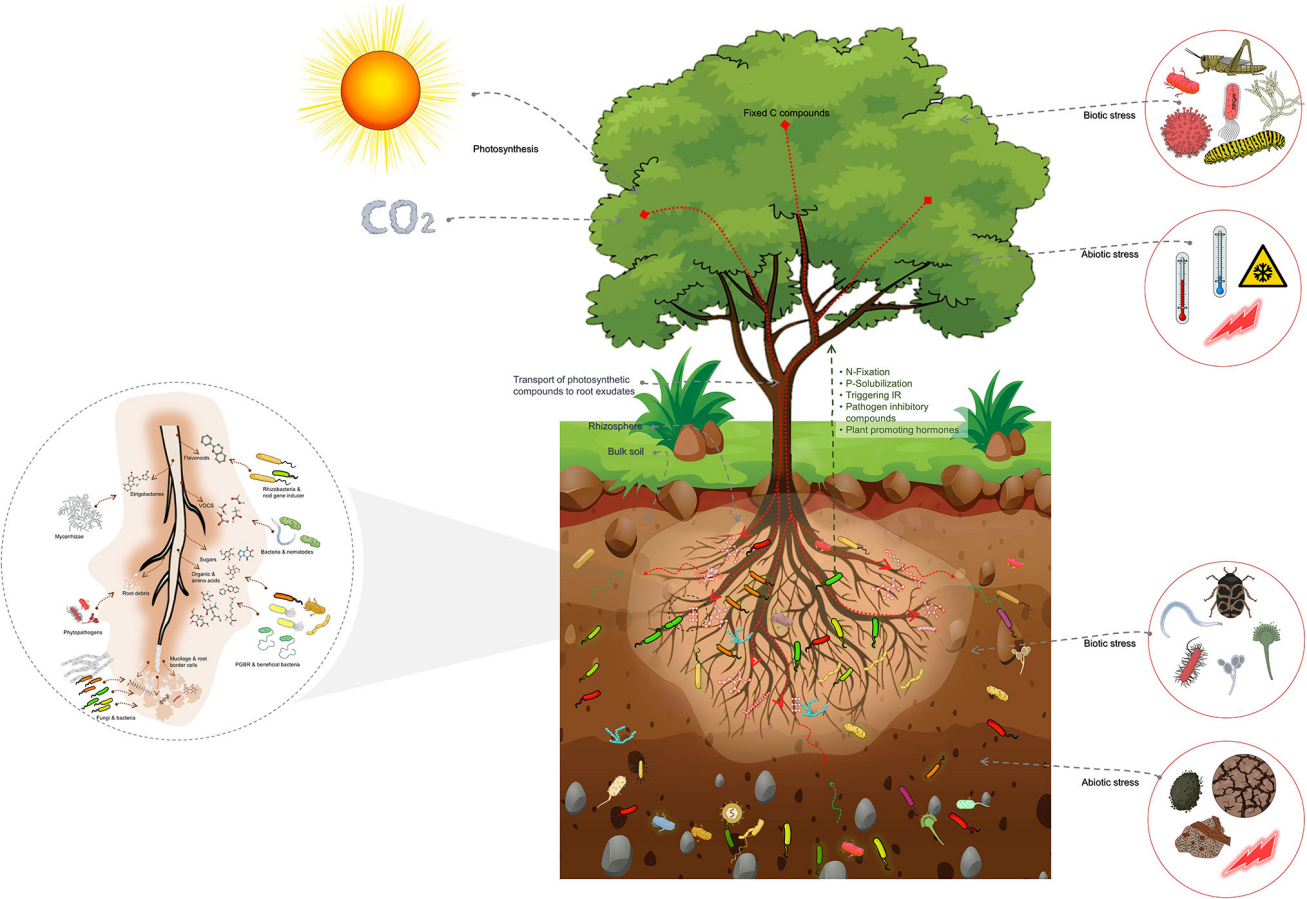
Schematic representation of the host plant-mediated assembly of the rhizosphere microbiome, emphasizing the importance of root exudates in attracting specific microbial groups from the bulk soil. Root exudates comprise a substantial proportion of plant photosynthetic compounds transported into the roots. These exudates are crucial in the formation of the rhizosphere microbiome. The bulk soil comprised of a highly diverse array of microbial groups serves as the source from which the rhizosphere microbiome is selected. The rhizosphere microbiome is abundant in copiotrophic microbial groups, which flourish in environments with ample nutrient availability. Additionally, the figure highlights the impact of biotic and abiotic factors above- and below-ground on the assembly of the rhizosphere microbiome. These factors trigger physiological changes that lead to changes in root exudates and subsequently influence the assembly of the rhizosphere microbiome. These factors also shape the composition and diversity of the rhizosphere microbiome, influencing plant growth and health.

The impact of host plants on the composition of the rhizosphere microbiome is through the release of root exudates, with different types of exudates attracting different groups of microbes (Figure 2).

#### 2.1.2. Plant developmental stages

The root exudates undergo shifts in response to exogenous stimuli by the pathogenic attack, as explained above, and in response to endogenous stimuli during the plant developmental stages. During the early plant developmental stage at the onset of germination, the seed (spermosphere)-associated microbiota, which are generally host-specific, contributes significantly to the assembly of rhizosphere microbiome composition mainly by enriching specific microorganisms that are useful for germination and healthy growth of the plant (Shao et al., 2021). The dynamic interactions between the germinating seeds, their associated spermosphere indigenous microbiota, and the surrounding soils are pivotal for plant growth and development. They are the first contact between the plant and soil microbiota (Nelson, 2004; Adam et al., 2018). The microbial composition of the spermosphere is mainly host-specific and comprised of unique composition, as the microbial population is mainly regulated by the carbon depositions of the seeds during germination along with the influence of soil biotic and abiotic properties (Nelson, 2004; Schiltz et al., 2015). Germination is initiated by seed imbibition, in which the influx of water into dry seeds is increased by the physical activity of the seed's chemical composition. Subsequently, the seed exudates that include proteins, lipids, and carbohydrates rapidly leak into the spermosphere (Koizumi et al., 2008). Along with the leakage of exudates containing seed components, other metabolites and low molecular weight compounds, including fatty acids, amino acids, phenolics, and volatiles, are exudated by the germination radicle and act as the primary driving force in the regulation of the microbial assembly of the spermosphere (Schiltz et al., 2015).

The spermosphere microbiota could therefore be considered the main building block for the rhizosphere microbiome structure, along with the soil microbial composition. Roberts et al. (2009) provided evidence that the metabolic activity of microbes in the spermosphere is dependent on the host plant and the specificity of the seed exudates. The authors described that the population and metabolic activity of *Enterobacter cloacae* were increased in pea plants compared to cucumber plants. Other studies argued that plant species only have a limited impact on the microbial community compared to the moisture content, with biotic and abiotic conditions around germinating seeds proposed as the main factors influencing the microbial composition of the spermosphere at this stage (Singh et al., 2009; Ofek et al., 2011; Schiltz et al., 2015). The collective evidence indicates that both factors have an undeniable influence on the microbial composition of the spermosphere, although in certain cases, soil factors could overcome the influence of host genotype or could suppress certain indigenous microbial groups. Of note, the spermosphere could include or attract phytopathogenic members, which might result in the development of disease under specific circumstances that favor the growth of the pathogens over other beneficial microbes (Torres-Cortés et al., 2019).

Following germination and during later plant developmental stages, other events significantly influence the rhizosphere microbiome composition. A study on the root exudates of rice at different growth stages reported that root exudation was low at the seedling stage and increased during flowering, with a subsequent decrease during maturation. The authors described that the composition of root exudates varied significantly between different growth stages, as the exudation of sugars was substituted with organic acids with advancing plant growth (Aulakh et al., 2001). Another study on the root exudates of *Arabidopsis* at different developmental stages during the lifespan concluded that the process is genetically programmed following the developmental pattern (Chaparro et al., 2013). The composition of root exudates varied significantly based on the developmental stage as the levels of sugars and sugar alcohols were higher in early stages and decreased through development, while the levels of amino acids and phenolics increased over time. Notably, there was a significant positive correlation between the functional capacity represented as microbial functional genes related to the metabolization of corresponding compounds in the rhizo-microbiome and the root exudation pattern, confirming the strong link between and prompt response between the physiological status of the host plant and the below-ground microbial world (Chaparro et al., 2013). In another study, a systematic proteomic analysis of the *Arabidopsis* root exudates throughout plant development revealed that defense-related proteins, such as chitinases, glucanases, and myrosinases, were secreted by the roots at the flowering stage (De-la-Peña et al., 2010). These observations could explain the increased resistance to pathogens during the transition to vegetative and flowing stages as shown in *Arabidopsis* and other plants, and the link between plant-related above-ground events (e.g., flowering) on the root exudates and consequently on the rhizobiome composition (Neale et al., 1990; Samac et al., 1990; Aulakh et al., 2001; De-la-Peña et al., 2010). Further confirmation of the genetic regulation of plant root exudates to support plant defense during certain developmental changes is evident during the development of lateral roots. During lateral root emergence, these sites are more vulnerable to pathogenic attacks that could have more detrimental effects on plants due to the breakage of the epidermis by emerging roots; hence, the defense exudates are enhanced (Baetz and Martinoia, 2014). Benzoxazinoid compounds are produced by maize during and immediately after lateral root and crown root emergence (Park et al., 2004; Guo et al., 2016).

**Figure 3:**
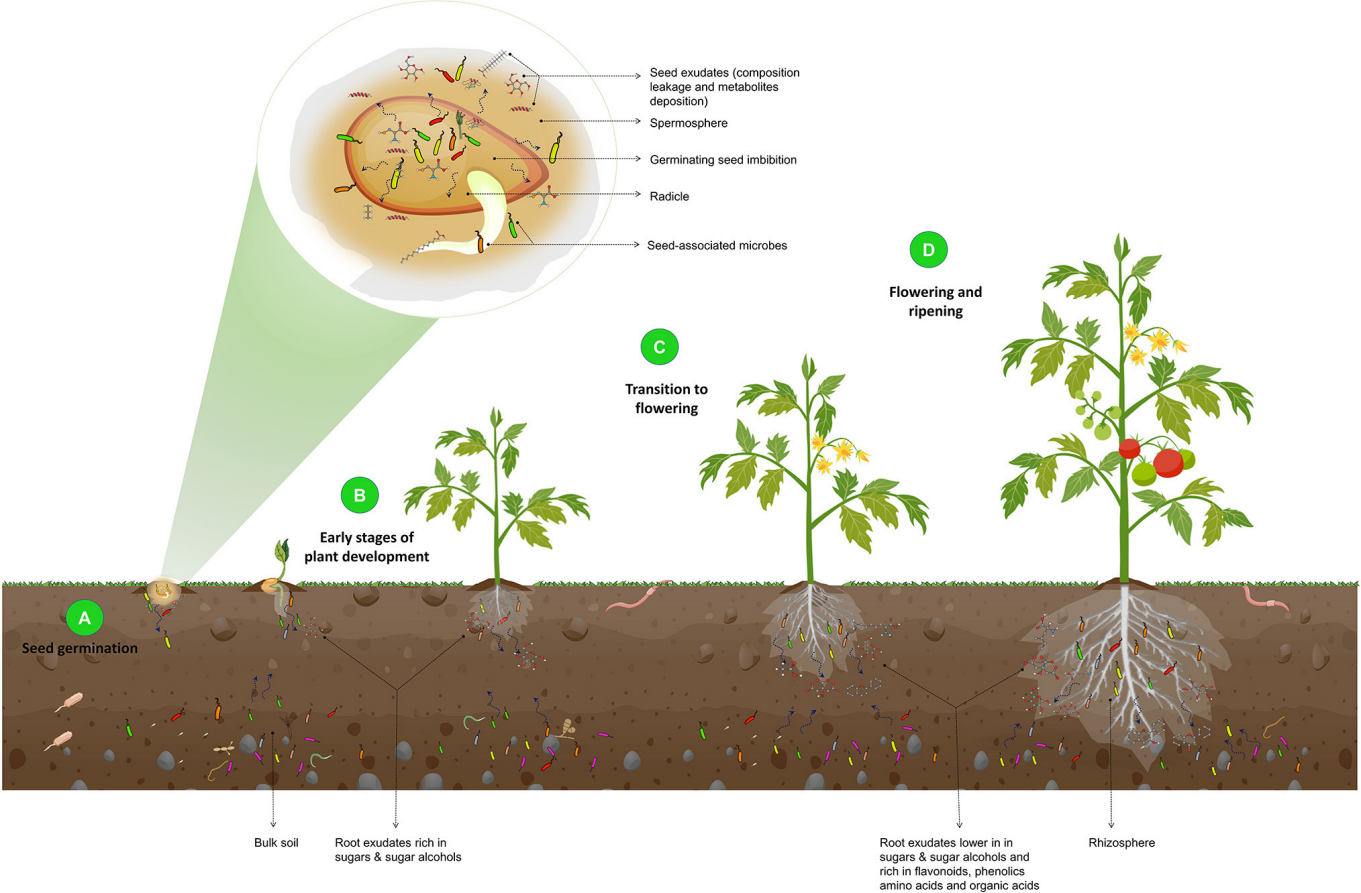
Illustration of the effect of plant developmental stages on the composition of the rhizosphere microbiome. During seed germination **(A)**, an increase in seed water content leads to the leakage of seed contents and the release of metabolites and exudates, such as fatty acids, phenolics, amino acids, and volatiles. These exudates, along with the seed epiphytic and endophytic microbiota, are released into the spermosphere and serve as the foundation for the rhizosphere microbiome and the initial point of interaction with the soil. In the early stages of plant development **(B)**, root exudates that are rich in sugars and sugar alcohols attract basic rhizosphere microbiota. As the plant transitions from the vegetative stage to flowering **(C)** and ripening **(D)**, root exudates shift to lower sugars and sugar alcohols and increased flavonoids, phenolics, amino acids, organic acids, and defense-related proteins. As a result, beneficial microorganisms are recruited. They protect the plant from pathogenic attacks, as this is a critical stage of growth in which plants are particularly vulnerable to phytopathogens.

The rhizosphere microbiome assembly and the influence of the plant developmental stages on the microbial composition are illustrated in Figure 3.

#### 2.1.3. Plant species and genetic makeup

As described above, plant tissues and the specificity of rhizodeposition are the driving forces for the selection of microbial composition in the rhizosphere. Therefore, the plant genetic makeup largely controls the composition of the rhizosphere microbiome (Berendsen et al., 2012). Although environmental factors and soil properties may have a significant impact on the microbial composition of the rhizosphere, specific plant species have relatively distinct microbial compositions (Berg and Smalla, 2009; Li et al., 2018). Supporting evidence has been provided by studies of the rhizosphere microbial composition of specific plants in different regions and environments (Bouffaud et al., 2014; Ofek-Lalzar et al., 2014; Matthews et al., 2019). In this perspective, a significant correlation was observed between the genetic distance of the rhizosphere microbial communities with the phylogenetic distance of the host plant genotype, indicating the influence of the evolutionary history of a particular plant genotype on the selection of bacterial taxa and shaping of the rhizosphere microbiota (Bouffaud et al., 2014). In wheat, maize, rape, and barrel clover, the root exudates can significantly influence the structure of the rhizosphere bacterial community when grown separately in the same soils (Haichar et al., 2008). Jiang et al. (2017) reported that cultivars of blueberry significantly affected the diversity and complex co-occurrence networks of rhizosphere microbiota, particularly the keystone bacterial species, with crucial roles in nutrient dynamics in the rhizosphere.

In addition to the difference in composition, the functional capacity is evident as the genetic background for specific functional traits needed for plant fitness or the ecosystem can be selectively enriched as a function of the host plant. A clear example is forest ecosystems, in which plant species promote a more rapid soil decomposition of a litter of their own species rather than the litter of other plant species due to the host-specialization of the soil and rhizosphere microbiome to the above-ground plant (Ayres et al., 2009). Another example of the regulation of below-ground functional capacity through plant root exudates is rice plants, in which the production and emission of methane are regulated by rice root exudates that vary significantly based on the cultivar and developmental stage (Aulakh et al., 2001). Yan et al. (2017) suggested that the rhizosphere selects specific species based on functional traits that are relevant for interactions with the plant and not necessarily restricted to the particular taxonomic group. In another study, the functional capacity, represented as functional gene expression, was enriched in the rhizosphere, as confirmed by meta-transcriptomic analysis (Ofek-Lalzar et al., 2014). Together, these results provide evidence that functional traits could be considered targets of the assembly of the rhizosphere microbiome and could explain the wide variability in the studies reporting the taxonomic differentiation of the rhizosphere microbiome composition. This is due to the significant functional redundancy present within the soil microbiome, which can be viewed as ecological insurance for maintaining functionality in the event of potential induced shifts, or selective activity by above-ground plants (Mendes et al., 2015).

### 2.2. Soil abiotic factors and rhizosphere microbiome assembly

#### 2.2.1. Soil types and properties

Along with the roles of the host plant genotype and the rhizodeposits, soil physicochemical properties are also important in shaping the microbial composition in the rhizosphere. Edaphic factors greatly influence the microbial diversity in bulk soils that are considered the microbial seed bank for rhizosphere microbiome assembly (Lennon and Jones, 2011). A continental-scale study of soil bacterial communities across North and South America revealed the pronounced influence of soil pH on soil microbial diversity (Fierer and Jackson, 2006). The authors described that soil microbial diversity was largely independent of geographic distance, while soil pH was a key driver to the microbial composition as the neutral soils displayed higher microbial diversity than acidic soils. Similarly, Griffiths et al. (2011) conducted a multi-scale spatial assessment of soil bacterial community profiles across Great Britain and found that bacterial diversity was strongly correlated with soil pH. In another continental-scale study on the co-occurrence patterns of rhizosphere microbiomes in soybean plants in China, soil pH, among other environmental variables, was found to be the strongest predictor of bacterial network geographical distribution. Specifically, a large proportion of the network links were associated with Acidobacteria, which are largely influenced by soil pH (Zhang et al., 2018).

In the rice paddy environment, Guo et al. (2022) reported that pH was the major driver of the rhizosphere microbial network and that higher pH levels were associated with greater stability and complexity, potentially leading to increased efficiency in nutrient cycling. This effect is likely related to the influence of pH on the taxonomic composition of microbial species. For instance, Verrucomicrobia, Acidobacteria, Chloroflexi, and Planctomycetes were more abundant in low pH rhizosphere soils, whereas Actinobacteria, which are involved in organic matter decomposition, were more abundant in relatively higher pH rhizosphere soils.

Soil type was also found to profoundly influence the structure of functional microbial communities as compared to the influence of plant cultivars in the rhizosphere of potato plants (Inceoglu et al., 2012). The same influence of the soil physicochemical characteristics was observed for strawberry plants on the mycorrhizal colonization level and assembly structure, with no difference noted between plant cultivars (Santos-González et al., 2011). Under field conditions, the influence of soil type on the composition of the rhizosphere microbial community was confirmed for different soils exposed to identical cropping history and the same climatic conditions for more than 10 years. A distinct microbial community composition that was specific for the soil type was still displayed, regardless of the prolonged cultivation period (Schreiter et al., 2014). The influence of soil type goes beyond influencing the rhizosphere microbial communities. In one study, soil type defined the composition of root-inhabiting endophytes in *Arabidopsis*. The authors detected soil-type-specific microbes within the root endophytic microbial assemblies and described the limited, mainly quantitative and ribotype-differentiative, effect of the plant genotype on the root endophytes profile (Bulgarelli et al., 2012). Even in the desert environment, local soil characteristics, mainly soil pH and carbon content, were the primary driver of microbial diversity in the bulk soil and rhizosphere (Andrew et al., 2012).

#### 2.2.2. Drought and environmental stress

Soil abiotic factors, such as farming systems, agricultural practices, and different forms of stress, including drought, can also significantly influence the rhizosphere microbial composition. Under drought stress, structural and functional adaptations are generally observed in soil and rhizosphere microbial communities. A more profound drought-induced shift was described in the rhizosphere microbial communities compared to the bulk soil. The microbial communities in the rhizosphere are exposed to the direct influence of water scarcity and also to the indirect influence of physiological and biochemical alterations in the host plant (Naylor and Coleman-Derr, 2018). The main influence on the rhizosphere microbial communities includes the reduction of fast-growing gram-negative bacterial taxa and enhancement of slow-growing gram-positive oligotrophic microbial taxa, such as those belonging to Actinobacteria and Firmicutes, which support plant growth during stress and mitigate the influence of drought stress (Fuchslueger et al., 2016). Several studies have confirmed similar findings in different host plants. For example, in rice, drought was found to cause a significant divergence in the microbial composition of the rhizosphere, endosphere, and bulk soils, with increased prevalence of Actinobacteria and Chloroflexi, and reduction of several Acidobacteria and Deltaproteobacteria (Santos-Medellín et al., 2017). In another recent study on different wheat cultivars, drought stress in combination with the soil type and farming system influenced the rhizosphere microbiome structure and functional capacity. More adaptive microbial communities developed, as the drought-tolerant taxa along with the enzymatic activity and carbon degradation-related genes increased in response to drought (Breitkreuz et al., 2021). The increased functional capacity for carbon degradation observed in drought-affected soils corresponds to the lower carbon availability in dry soils and the need for the degradation of plant complex carbohydrates (Bouskill et al., 2016).

The overall changes in the rhizosphere microbial composition under drought stress could be considered a rapid community-scale adaptation to the changing environment that participates in sustaining plant growth under harsh conditions. In this context, soil microbial communities that have been previously exposed to drought supported plant fitness and promoted survival under drought stress when associated with plants (Lau and Lennon, 2012). The roles of the rhizosphere microbial communities in alleviating drought stress to plants include the enhanced production of phytohormones, such as indole-3-acetic acid and abscisic acid. These phytohormones promote plant growth by improving photosynthetic activity and enhancing adventitious root development for more effective use of water (Gowda et al., 2011; Cohen et al., 2015). Moreover, under drought stress, plants produce ethylene to maintain homeostasis, which affects the growth of roots and shoots. Certain rhizobacteria produce 1-aminocyclopropane-1-carboxylate (ACC) deaminase, which inhibits the ACC ethylene precursor in plants and thus contributes to sustaining plant growth under stress conditions (Mayak et al., 2004; Arshad et al., 2008).

In addition to drought, other forms of stress could influence the assembly of rhizosphere microbial communities and produce specific compositional shifts that might play a role in alleviating the deteriorative effect on plant growth. These stresses include salinity, heavy metals, water logged condition, residues of pesticides, and heat. In a recent study by Tiziani et al. (2022), targeted and untargeted metabolomics were used to evaluate the effect of different forms of drought alone, heat stress alone, and both in combination on the root exudates of maize and the links between stress-affected root exudates and rhizosphere microbial composition. Root exudates significantly changed based on the type of stress. These stress-specific exudates influenced specific microbial taxa that could be beneficial to plants. In addition, several plant growth-promoting bacteria and fungi enriched under stress conditions could increase host plant tolerance.

On the forest scale, the Korean fir tree (*Abies koreana*), an endangered tree species, has declined in prevalence for as-yet unknown reasons. *A. koreana* has been extensively studied to explore the causes of their decline and to suggest possible intervention strategies to preserve the tree population (Koo et al., 2017; Seo et al., 2021). Abiotic factors, including moisture imbalance, heat stress, precipitation rates, heavy winter snow, and vegetation changes, might be the causes of the decline (Ahn and Yun, 2020). Han et al. (2022) observed dysbiosis in the rhizosphere microbiome composition between standing dead and healthy trees. The diversity and richness were significantly reduced in the declining trees. More specifically, several microbial taxa, including *Bradyrhizobium, Rhizomicrobium, Caulobacter, Nitrosospira, Rhizobacter, Paraburkholderia, Rhizobium, Devosia, Caballeronia, Niveispirillum, Dyella, Herbaspirillum, Frankia, Streptomyces, Actinoallomurus, Lysobacter, Luteibacter, Mucilaginibacter*, and *Variovorax*, that could play roles in alleviating the impact of abiotic stress on the trees were more abundant in the rhizosphere of healthy trees compared to declining trees. These findings suggest the potential involvement of the rhizosphere microbiome in protecting forest trees from the changing climate and paving the way for further investigations to save endangered trees.

### 2.3. Microbial roles in rhizosphere microbiome assembly

#### 2.3.1. Soil beneficial microbes influence rhizosphere microbiome assembly

As described above, the composition of root exudates and secretion pattern largely depends on plant-related factors. They have the main roles along with soil type and physicochemical properties in shaping the rhizosphere microbial composition. Intriguingly, the microbial composition in the rhizosphere also has an important role in regulating plant root exudates and consequently the community assemblage. Several reports in the literature have described the influence of rhizosphere microbes on root exudates. *Pseudomonas putida*, a well-known rhizosphere microbe and plant resistance inducer, has a significant impact on the composition of *Arabidopsis* root exudates (Matilla et al., 2010).

Another remarkably influential microbial group in the rhizosphere is arbuscular mycorrhizal fungi (AMF). These fungi significantly influence the regulation of plant root exudates. Plants recruit and enhance the colonization with AMF through root exudates, mainly flavonoids (Jones et al., 2004; Tian et al., 2021). In return, AMF specifically alters the composition of root exudates, including changes in the number of total sugars, compositional alterations of organic and amino acids, and increased levels of nitrogen compounds, phenolics, and gibberellins (Jones et al., 2004; Hage-Ahmed et al., 2013). Such AMF-mediated quantitative and qualitative alterations in the root exudates have been linked to consequent beneficial changes in the rhizosphere microbial composition, including the suppression of pathogenic microbes and enhancement of plant growth-promoting rhizobacteria (PGPR) (Norman and Hooker, 2000; Gupta, 2003; Hage-Ahmed et al., 2013). In another study on pine seedlings, root-associated ectomycorrhizal fungal communities significantly influenced root exudation rates (Meier et al., 2013). These collective findings link the presence of certain microbial taxa that are beneficial for plants to particular changes in the rhizosphere microbial composition assembly, whether directly by association and promotion with other microbial groups and antagonism to others, or indirectly by causing shifts in the plant root exudation pattern and consequent shifts in the rhizosphere microbial assembly.

#### 2.3.2. Soil-borne phytopathogens influence the rhizosphere microbiome

Phytopathogens trigger particular changes in root exudates as a method of plant defense. Several studies described that both below- and above-ground phytopathogenic attack on plants results in a shift in the composition of rhizosphere microbial communities (Li et al., 2021). Plants can recruit particular microbial taxa that can antagonize soil-borne pathogens upon infection, as a defensive strategy. In a study utilizing microbiome network analysis, certain microbial taxa, such as those belonging to Pseudomonadaceae, Chitinophagaceae, and Flavobacteriaceae families, were closely associated with wheat rhizosphere affected with root rot and bare patch disease caused by *Rhizoctonia solani* (Poudel et al., 2016). The microbial taxa found in association with diseased plants in the latter study are generally copiotrophs that are enriched in response to the increased exudation by diseased plants, and the other cell wall and cytoplasm components of damaged roots in the presence of the pathogen. Among these enriched microbial taxa, *Chitinophaga* might consume the chitin of the fungal biomass and *Chryseobacterium soldanellicola* has been reported with biocontrol activity against *R. solani* (Yin et al., 2013). In another study, the root exudates of *Ralstonia solanacearum*-infected tomato plants showed a specific different composition compared to the composition of healthy plants, with changes in phenolic compounds and a significant increase in caffeic acid (Gu et al., 2016). In the latter study, exudates from *R. solanacearum* infected plants suppressed the pathogen and resulted in the development of different microbiome communities, indicating both direct and indirect control activities on the pathogen. Such phytopathogenic attacks on plants have also been found to cause drastic shifts in the rhizosphere microbial community composition and functional capacity.

#### 2.3.3. Impact of above-ground disease-causing organisms on rhizosphere microbiome composition

The above-ground phytopathogenic attacks can cause significant shifts in the below-ground rhizosphere microbial community composition, even without direct competition, via an indirect influence on the plant physiology and root exudate pattern (Li et al., 2021). In this context, Trivedi et al. (2012) reported the significant impact of *Candidatus* Liberibacter asiaticus (the obligate endophytic bacterium that causes the destructive Huanglongbing disease on citrus trees) on the microbiota composition of the tree rhizosphere. The evident impact of *Ca*. L. asiaticus on the below-ground microbial composition and functional potential is linked to the strong influence of the phytopathogen on the petitioning of photo-assimilates and the changes in root exudation. Several studies involving the model plant *Arabidopsis thaliana* have confirmed the involvement of above-ground pathogenic infection in manipulating the rhizosphere microbial community. The finding reflects the influence of the root exudate pattern for the recruitment of specific beneficial microbes, which enhances plant defense against infection. Rudrappa et al. (2008) demonstrated that foliar infection with the phytopathogen *Pseudomonas syringae* triggers the secretion of malic acid in the roots. The secreted malic acid acts as a stereoselective chemical signal to selectively recruit and promote root colonization by the beneficial *Bacillus subtilis* that induces plant systemic resistance, which restricts pathogen multiplication and enhances plant defense against attack. In another study, leaf infection with *Hyaloperonospora arabidopsidis*, the airborne biotroph causal agent of downy mildew, resulted in a change in the *Arabidopsis* rhizosphere microbial composition by specifically promoting a consortium of bacteria composed of three bacterial species (*Microbacterium, Stenotrophomonas*, and *Xanthomonas*). The three enriched bacteria were isolated and demonstrated to synergistically colonize the *Arabidopsis* roots to promote growth and induce systemic resistance against downy mildew. The infection in the first plant population increased the resistance against the same pathogen in subsequent plants grown in the same soils, confirming the soil legacy effect (Berendsen et al., 2018).

Insect infestation has also been associated with significant shifts in the rhizosphere microbiome composition. In a study of above-ground phloem-feeding whiteflies, Kong et al. (2016) reported that infestation caused a dramatic transition in the rhizosphere microbiome composition. As evident with the phytopathogens, the shifts of the rhizosphere microbiome composition upon insect infestation tend to target the enrichment of specific microbial groups that support plants under attack. In the case of whitefly infestation, specific microbial taxa (e.g., *Pseudomonas*) rapidly increased in relative abundance following whitefly infestation, which could contribute to plant protection (Saravanakumar et al., 2007; Kong et al., 2016). The observed shifts in the rhizosphere microbiome composition could be a result of the previously reported changes in the root exudation as a result of insect infestation (Park and Ryu, 2014; Song et al., 2015). On the forest scale, above-ground infestation with the pine wood nematode, *Bursaphelenchus xylophilus*, which causes destructive pine wilt disease, can affect the rhizosphere microbiome composition; a clear distinction was observed between the rhizosphere microbiome profile of nematode inoculated trees compared to un-inoculated control (Han et al., 2021). The authors described that the rhizosphere of pine trees showing wilting symptoms had lower levels of several microbial taxa, including *Paraburkholderia, Bradyrhizobium, Rhizobacter, Lysobacter*, and *Caballeronia*. All promote plant growth and could play a role in the protection of healthy plants from nematode invasion (Han et al., 2021). It can be inferred that phytopathogens and parasites should not only be viewed from their direct deteriorative influence on different plant tissues but also based on their dysbiosis influence on the rhizosphere microbiome.

#### 2.3.4. Microbe-mediated plant-to-plant signaling influence distal rhizosphere microbiome

The more we understand about the complex interaction between and among plants, beneficial microbiota, and pathogens, the more we realize the sophisticated levels of organization and synchronization within plant ecosystems. The intriguing findings suggesting the possible signaling between neighboring plants upon pathogenic infection could be seen as evidence of co-evolutionary crosstalk synchronization. In this scenario, upon infection with a pathogen (under attack), a plant produces messenger alerting molecules (volatiles) to neighboring plants. The latter plants receive the alerting signals and respond by assembling a defensive rhizosphere microbiota for protection against the pathogen. This scenario is supported by several results, confirming the hypothesis of plant distal rhizosphere microbiota recruitment (Heil and Ton, 2008; Li et al., 2021). Several studies have confirmed that plants release volatile organic compounds (VOCs) upon attack with a pathogen (microbe-induced plant volatiles) or insects (herbivore-induced plant volatiles) (Cellini et al., 2018; Sharifi et al., 2018). VOCs that include the bioactive hexanal isomers and 2,3-butanediol produced by apple plants during infection with the phytopathogens *Erwinia amylovora* and *Pseudomonas syringae* pv. *Syringae* trigger the induction of resistance by the activation of salicylic acid synthesis and signal transduction in neighboring uninfected healthy plants. The result is the suppression of phytobacterial growth and migration in plant tissues (Cellini et al., 2018). More recently, Kong et al. (2021) provided a model example using tomato plants of specific plant-to-plant VOC signals via β-caryophyllene. β-caryophyllene is released upon application with a model PGPR *Bacillus amyloliquefaciens* in one plant. When the released molecule is received by a spatially separated neighboring plant, salicylic acid-mediated resistance is induced. The induction results in specific shifts in the rhizosphere microbiome composition that was surprisingly similar to the original PGPR-treated tomato (transmitter) plant.

**Figure 4:**
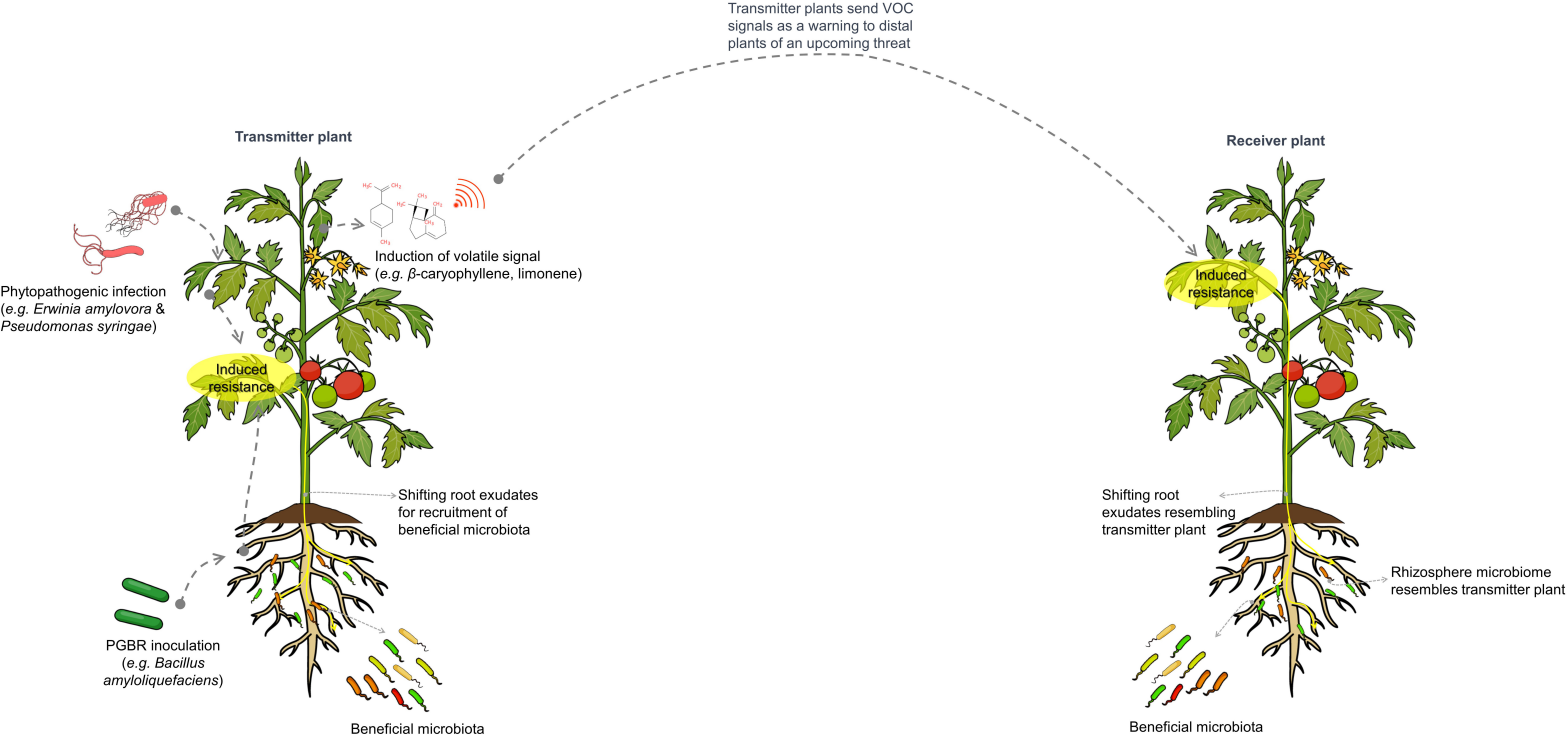
Schematic representation of microbe-mediated plant-to-plant signaling, which influences the composition and assembly of distal rhizosphere microbiomes. Plants under pathogen attack or treated with plant growth-promoting microbes will induce salicylic acid-mediated resistance and alter root exudates to recruit beneficial microbiota that supports the plant during infection. Notably, these plants also emit volatile signals, such as β-caryophyllene and limonene, which warn neighboring plants of expected pathogen invasion. This leads to the induction of resistance and changes in rhizosphere microbiomes similar to those of the transmitting plant.

The influence of microbe-mediated plant-to-plant signaling on the distal rhizosphere microbiome is illustrated in Figure 4.

Taken together, the rhizosphere microbiome assembly can be best summarized as a series of interconnected events involving multi-interactions between linked variables that include soil, host plant, associated microorganisms, and environmental factors. The original seed spermosphere microbes along with the soil microbes constitute the reservoir for the microbiome assembly. The host plant participates by rhizodeposition and constituent exudates that can have variable impacts depending on the genotype, physiological status, and developmental stages. All these are shaped by the prevailing above-ground and below-ground environmental conditions. Understanding the complex network of below-ground and above-ground interactions would lead to possible strategies to manipulate the composition of the rhizosphere microbiome for exploiting the beneficial traits of the plant.

## 3. Rhizosphere microbiome engineering to enhance plant growth and tolerance to biotic and abiotic stresses

**Figure 5:**
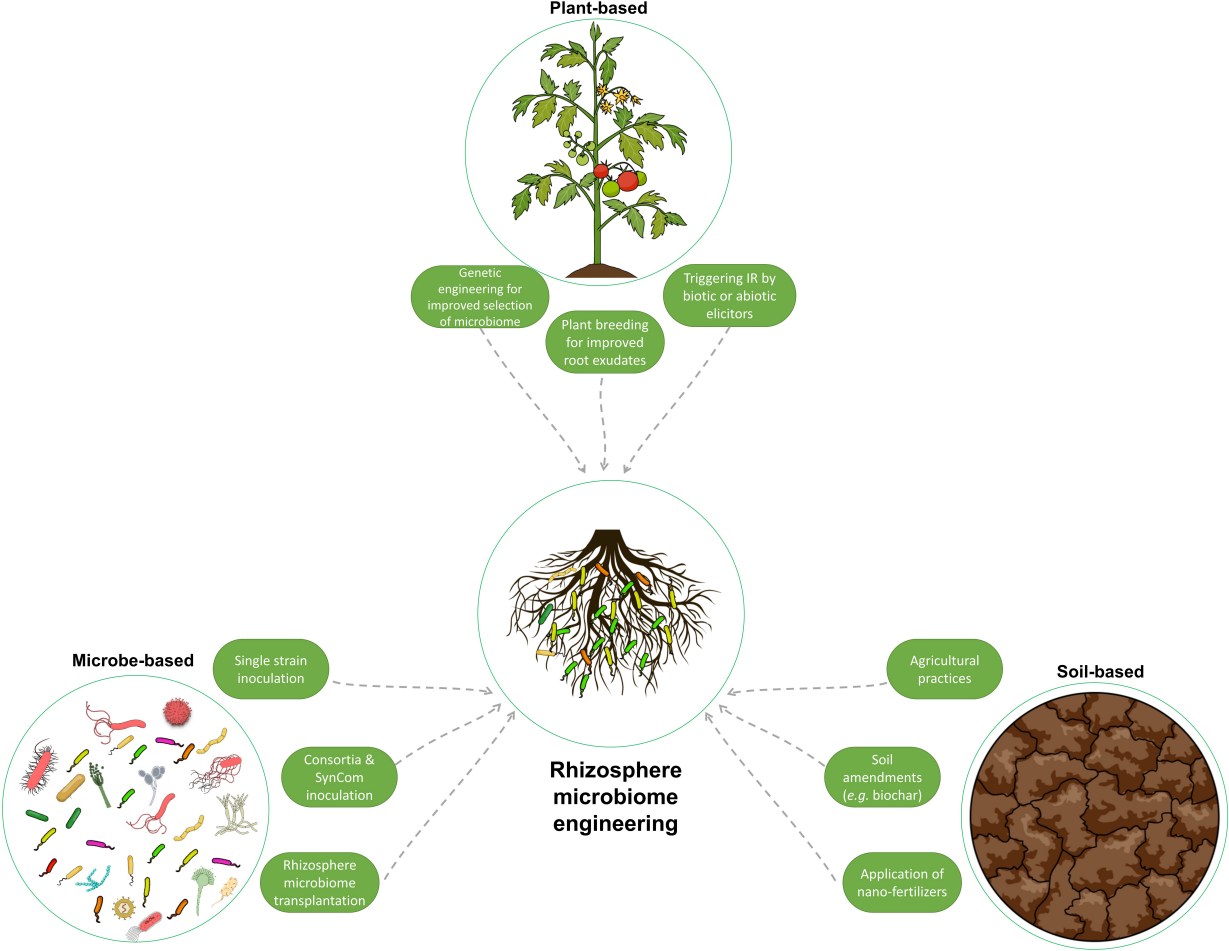
Illustration of the various methods used to manipulate and engineer the rhizosphere microbiome to enhance plant growth and resilience to stress. These methods can be classified as host plant-mediated, soil-mediated, and microbe-mediated. They range from traditional techniques, such as soil amendments, to more advanced methods, such as genetic engineering and rhizosphere microbiome transplantation. This figure summarizes the different strategies used to improve the composition of the rhizosphere microbiome.

As discussed in the previous section, establishing the rhizosphere microbiome composition is regulated by several variables, mainly host-, soil-, and microbe-related. In this section, the traditional and modern strategies that have been applied or have the potential to manipulate the rhizosphere microbiome composition to confer beneficial traits, such as supporting plant growth and tolerance to biotic and abiotic stress, are discussed (Figure 5).

### 3.1. Plant-based approaches

In the host plant-based approaches, understanding the regulatory roles of the host plant on the rhizosphere microbiome and the above- and below-ground crosstalk, mainly via root exudates and rhizodeposits, as explained in the previous section, has been investigated. The aim is to exploit the plant's ability to recruit specific microbes, which will contribute to the enhancement of plant fitness and tolerance to stress.

#### 3.1.1. Rhizosphere microbiome manipulation via external elicitors triggers plant physiological changes

Plant manipulation aimed at enhancing rhizosphere microbiome composition and functionality could be achieved by triggering physiological changes via external elicitors. Treatments with resistance-inducing elicitors trigger particular plant physiological changes, such as increasing the pathogenesis-related (PR) proteins, phenolics, alkaloids, chitinases, and other hydrolytic enzymes that contribute to enhancing plant defense against pathogens, parasites, and insects (Zaynab et al., 2018; Abdul Malik et al., 2020). It has been suggested that such plant physiological changes will be reflected in variations of the root exudates and will influence the microbiome composition. Mannaa et al. (2020) reported that the use of foliar treatment with resistance-inducing chemical elicitors, including methyl salicylic acid and acibenzolar-s-methyl, for the induction of resistance of pine trees against pine wilt disease was associated with significant positive changes in the rhizosphere microbiome composition. Specifically, the relative abundance of several microbial taxa with essential ecological and plant growth-promoting roles was increased. These included members of the *Devosia, Bradyrhizobium, Mesorhizobium*, and *Hyphomicrobium* genera. Among the observed changes in the rhizosphere upon foliar treatment with the resistance-inducing elicitors, there was a notable reduction in the chitinolytic microbial taxa, including *Paenibacillus* and members of the Chitinophagaceae family, such as *Nemorincola* and *Rurimicrobium*. These reductions might be connected with the increase in plant exudation of chitinase, which degrades chitin in the rhizosphere and reduces the available substrate for the growth of such microbial taxa (Mannaa et al., 2020).

A similar trend was observed when microbial resistance-inducing elicitors were used in the foliar treatment of pine trees against pine wilt disease. Han et al. (2021) reported the induction of systemic resistance in pine trees against pine wilt disease upon foliar treatment with the biocontrol *Pseudomonas koreensis* and *Lysobacter enzymogenes*, which resulted in particular changes in the rhizosphere microbiome composition. The changes were evident as increased relative abundances of specific microbial taxa that included *Nitrospirillum, Bacillus, Luteibacter*, and *Bdellovibrio*. These taxa might contribute to pine tree defense. It has been previously reported that foliar treatment with biocontrol bacterial agents against pine wilt disease results in differential expressions of defense-related genes, including PR genes b-1,3-glucanase, class I chitinase, class IV chitinase, thaumatin-like protein, peroxidase, ribonuclease-like protein, antimicrobial peptides, and metallothionein-like protein. The changes likely contribute to the alterations of the root exudates and may explain the observed changes in the rhizosphere microbiome composition (Kim et al., 2019). Thus, the tight link between the above- and below-ground microenvironment could be utilized by the manipulation of plant physiology by chemical or biological resistance-inducing elicitors to improve the composition of the rhizosphere microbiome.

#### 3.1.2. Plant breeding-based manipulation of the rhizosphere microbiome

Another attractive approach for plant-mediated manipulation is the integration of plant impact on rhizosphere microbiome within plant breeding programs aiming for cultivars with specific root exudates that encourage beneficial microbiome composition and functions while discouraging phytopathogens (Wissuwa et al., 2009; Bakker et al., 2012). Research on the involvement of rhizosphere microbiome-associated traits in plant breeding investigations is very limited. Research has been hindered by the complexity and variability among rhizosphere microbiomes associated with different environments, soil types, and microbial communities (Bakker et al., 2012). The concept of selection of plant cultivars related to specific regulatory traits for the rhizosphere microbiome has been confirmed. For instance, Badri et al. (2009a) described that a specific mutation in a single gene involved in root exudates resulted in significant changes in the phytochemical composition. Increased contents of phenolic compounds and fewer sugars were observed compared to the wild-type plant. These compositional changes in the root exudates were associated with modifications in the rhizosphere microbiome composition, which promoted beneficial microbial taxa involved in nitrogen fixation and heavy metal remediation (Badri et al., 2009a).

More recently, during breeding for resistance in common bean (*Phaseolus vulgaris*) against *Fusarium oxysporum* (the cause of root disease), the rhizosphere microbiome composition and functional capacity shifted significantly between susceptible and resistant cultivars. The findings of the greater abundance in resistant cultivars of specific rhizosphere-competent bacterial taxa, such as pseudomonadaceae, bacillaceae, solibacteraceae, and cytophagaceae, indicate their roles in providing complementary protection against fungal infections (Mendes et al., 2018). These results were further supported by another study that featured meta-transcriptome data, community-level physiological profiling, and resistome analysis. The rhizosphere microbiomes of the resistant plants were functionally different with the enhancement of several traits involved in plant defense and protection of bacteria against oxidative stress induced by pathogen invasion (Mendes et al., 2019). These findings further confirm the suggestion that breeding for resistance has a role in regulating rhizosphere microbiome assembly and functional capacity. Breeding programs should consider targeting the link between plants and the rhizosphere in reinforcing plant defense, enhancing fitness, and promoting productivity.

#### 3.1.3. Genetic engineering-based manipulation of the rhizosphere microbiome

Engineering plants to produce specific exudate compounds that are exclusively consumed by specific microbes, and favor their establishment, is a concept that is gaining attention as a potential method to improve the rhizosphere microbiome composition. This process happens naturally in plants. The soil microbe *Agrobacterium tumefaciens* is the first-known genetic engineer of plants. This microbe manipulates the composition of plant exudates. The natural genetic manipulation of the plant host occurs by the transfer of discrete fragments of oncogenic DNA to the host plant's genome to produce specific exudate compounds (phytohormones and opines). These compounds are exclusively utilized by the bacteria as nitrogen, carbon, phosphorus, and sulfur sources, providing a competitive advantage over other soil or root endophytic microbes (Gelvin, 1998; Flores-Mireles et al., 2012). The *Agrobacterium*-genetic colonization of plants led to *Agrobacterium*-mediated genetic transformation. This technique has been a dominant technology for the production of genetically modified transgenic plants for decades (Tzfira and Citovsky, 2006). Although naturally occurring, *Agrobacterium*-mediated genetic transformation results in the development of plant diseases, such as crown gall, the process provided insights that were investigated for the beneficial host-mediated manipulation of the rhizosphere composition. In the symbiotic interaction between the nitrogen fixing root nodulators *Rhizobium meliloti* and *Medicago sativa*, the opine-like rhizopine is produced in the root nodules by the *Rhizobium* induction. Rhizopine is then utilized as carbon and nitrogen sources. Thus, rhizopine can be considered a functional nutrition mediator in this plant–bacterial interaction (Murphy et al., 1987).

The discovery that only certain exudate compounds can be catabolized by specific desirable microbes has spurred the opine concept and created a “biased rhizosphere” in which certain microbial taxa are enhanced by the manipulation of the host plant to produce specific exudate compounds (Rossbach et al., 1994). Based on these pioneer findings, the concept of “rhizosphere engineering” based on the manipulation of the plant exudates has emerged as a potentially effective tool for recruiting specific desirable microbial taxa and triggering the expression of desired functional genes within the rhizosphere microbial communities. Rhizosphere engineering remained unexploited at that time due to the lack of knowledge of molecular techniques capable of studying the complex interactions within the rhizosphere microbiome (O'Connell et al., 1996).

Advancements in molecular tools and multiple meta-omics approaches have allowed a deeper understanding of community-based interactions at the molecular level. In addition, these advances have permitted studies of the composition and functional capacity of microbial taxa, including unculturable taxa. More precise investigations have targeted specific regulatory functions for rhizosphere microbiome engineering (del Carmen Orozco-Mosqueda et al., 2022). Geddes et al. (2019) established the transkingdom signaling by rhizopines produced from transgenic *Medicago truncatula* and *Hordeum vulgare* (barley) to rhizosphere bacteria. The established synthetic rhizopine-mediated signaling could potentially be utilized for targeted regulation of the composition and gene expression in the rhizosphere microbiome for the delivery of beneficial traits to plants (Geddes et al., 2019).

The first step toward the successful genetic manipulation of host plants for the regulation of the rhizosphere microbiome assembly is the accurate identification of the genetic elements controlling the selection and influencing the colonization by specific rhizosphere microbial taxa. Advanced molecular tools, such as genome-wide association (GWA) studies and quantitative trait locus (QTL), have been applied to map the potential genetic background for host plant selection of the rhizosphere microbiome. Deng et al. (2021), demonstrated the utilization GWA approach to identify specific genetic loci in different sorghum genotypes to control the selection and influence the colonization of particular rhizosphere microbial taxa. These findings facilitated the prediction of the rhizosphere microbiome structure of specific plant genotypes based on genetic information, even in different environmental conditions. More recently, Oyserman et al. (2022) used an integrated approach of quantitative genetics with the community-level microbiome, assisted by QTL analysis, to map the molecular features of the rhizosphere microbiome and the genomes of wild and domesticated tomatoes. The authors suggested that QTL-based investigations coupled with metagenomics could be utilized to identify the genetic basis for differential recruitment of the rhizosphere microbiome and target for improving the plant–rhizosphere microbiome interactions.

Advanced genome editing tools, such as zinc finger nucleases, site-specific transcription activator-like effector nucleases, and the CRISPR/Cas system, could be utilized for the manipulation of key genes in the plant genome involved in biosynthesis and metabolism, which are responsible for the recruitment of desirable rhizosphere microbes (Kumar and Dubey, 2020; Bano et al., 2021). In particular, the CRISPR-Cas9 system has been successfully applied in several previous studies for gene editing of plants for crop improvement, enhanced resistance, and metabolic pathways (Ortigosa et al., 2019; El-Mounadi et al., 2020). A recent study by Zhong et al. (2022) utilized CRISPR-mediated gene editing and *Agrobacterium rhizogenes*-mediated hairy root transformation in melon to reveal the involvement of a root-secreted metabolite (cucurbitacin B) in the selective enrichment of *Bacillus* and *Enterobacter* in the rhizosphere. These microbes confer resistance against the soil-borne fungal pathogen *Fusarium oxysporum*. The findings of this study provide applicable evidence of the targeted manipulation of plant genomes for improved plant fitness by the indirect influence on the rhizosphere microbiome composition. The findings also pave the way for prospective host-mediated rhizosphere microbiome engineering applications on other crops. The use of plant genome editing tools to target traits involved in rhizosphere microbiome assembly and plant–microbe interactions has immense potential and could contribute significantly to the next green revolution for crop productivity and stress tolerance.

### 3.2. Soil-based approaches

As described previously, soil and the related edaphic factors play a major role in the assembly of the rhizosphere microbiome composition and functionality. Thus, these factors could be a valid target to modify with the goal of manipulating the rhizosphere microbiome composition to enhance plant fitness. Agricultural practices including soil preparation and soil amendments, especially in organic systems, have been an integrated part of farming and planting for millennia, reflecting their considerable benefits for plant growth (Lori et al., 2017). The accumulated knowledge of the microbial elements of the soil and the rhizosphere has revealed that much of the beneficial effect of soil amendments is related to the direct or indirect influence on the microbial composition and the rhizosphere microbiome. Soil amendment with biochar is an old practice that dates back to early pre-Columbian Amerindian populations. The use of biochar has enhanced and sustained soil properties and fertility in tropical areas for approximately two millennia despite leaching due to heavy rains and intense weathering (Mann, 2002). A cultural-based study comparing the microbial community between historically biochar-amended soils and adjacent soils revealed significantly higher population, diversity, and richness in the amended soils (O'Neill et al., 2009). Other reports have also confirmed the influence of biochar amendment on the soil and rhizosphere microbial communities. Abujabhah et al. (2016) demonstrated the enrichment in microbial abundance by soil amendment with biochar and compost in an apple orchard. Significant shifts were observed in the rhizosphere microbiome of strawberries by the addition of biochar, with enhanced growth and disease resistance (De Tender et al., 2016).

Organic farming practices are generally linked to improved composition and activity of soil microbial communities (Luo et al., 2018). In a study comparing organic and conventional managed soils for the suppression of *Phytophthora* blight of pepper, organic soils displayed greater suppressive ability. This was mainly attributed to the differential assembly of the pepper rhizosphere microbiome with an increased abundance of an antagonistic *Bacillus* genus (Li et al., 2019). Visioli et al. (2020) linked the improved growth and quality traits of Tritordeum when grown under organic conditions compared to conventional farming conditions to the increased abundance of Bacteroidetes within the rhizosphere microbiome, which included several bacteria that were beneficial to plant and root growth. Organic farming practices, such as soil amendment with organic manure and lime, reportedly contributed to the amelioration of soil acidity and consequently improved the composition and activity of the rhizosphere microbiome by increasing the abundance of Actinobacteria and Proteobacteria, leading to enhanced suppression of soil-borne diseases (Chen et al., 2022). In another recent study comparing the long-term (18 years) soil management under organic and conventional systems on the soil microbial community, amendment with green and animal manure resulted in a shift of the microbiome to better suppressive fungal soil-borne pathogens (Khatri et al., 2023). The authors described an increased abundance of several antagonistic microbial taxa, especially those belonging to Acidobacteria, Proteobacteria, Bacteroidetes, Verrucomicrobia, and Gemmatimonadetes. The suppressive potential was confirmed in plant assays.

With the recent advancements in nanotechnology and the application of nanofertilizers as an efficient and eco-friendly alternative to conventional synthetic fertilizers, it has been reported that nanofertilizers can significantly influence plant-associated microbiota, including the rhizosphere microbiome (Raliya et al., 2017). The nanofertilizers provide a slow and sustained steady release of nutrients and aid in conserving the beneficial rhizosphere microbial community (Kalwani et al., 2022). In this context, soil amendment with conventional silicon and silicon nanoparticles reportedly enhances the microbial biomass in the rhizosphere and contributes to enhanced plant growth and fitness (Rajput et al., 2021). Another recent study of the maize rhizosphere microbiome reported the positive influence of nano-chitosan application on enhancing the bacterial population, particularly the beneficial plant growth-promoting groups, leading to enhanced plant growth (Agri et al., 2022). With certain nano-compounds, the antimicrobial activity could result in particular changes within the soil and the rhizosphere microbial communities. The antimicrobial effect of nanosilver was studied on the soil and rhizosphere microbial communities of maize plants (Sillen et al., 2015). The application of nanosilver resulted in a differential influence between bulk and rhizosphere microbial communities due to the strong rhizosphere–microbe association and interactions. The observed changes in the rhizosphere microbiome were apparently associated with increased benefits of the rhizosphere microbial community and reduced adverse plant properties as confirmed by the resulting overall increase in maize plant growth.

### 3.3. Microbe-based approaches

#### 3.3.1. Inoculation with individual strains

The direct introduction of bioinoculants is one of the most effective methods for improving the composition and functional capacity of the rhizosphere microbiome. These microorganisms (i.e., PGBR) support plant growth, antagonize pathogens, and maintain a healthy rhizosphere (Berendsen et al., 2012). They have been extensively studied and utilized as biofertilizers and biopesticides to enhance plant growth and resilience to both biotic and abiotic stresses by acting through a variety of mechanisms, such as plant nutrition, production of antibiotics, competition for nutrients, and induction of systemic resistance in plants (Berendsen et al., 2012; Pandit et al., 2022). The classical approach for the use of biocontrol bacteria in the rhizosphere is the isolation, characterization, and selection of effective strains. These strains are then used for single inoculation as biofertilizers to promote plant growth or against specific pests or diseases (Bulgarelli et al., 2012, 2015). Many studies have been performed to isolate and characterize biocontrol strains, such as those belonging to *Bacillus, Pseudomonas*, and *Streptomyces* (Tailor and Joshi, 2014; Hakim et al., 2021).

Along with the direct plant growth promotion or disease-controlling roles of the biocontrol agents, bioinoculants could positively influence the rhizosphere microbiome composition, leveraging the beneficial influence on plants. For instance, Xue et al. (2015) isolated *B. amyloliquefaciens* from suppressive soils and reported the biocontrol activity against Panama disease of banana caused by *F. oxysporum* f. sp. *cubense*. Treatment with the isolated biocontrol bacteria caused significant shifts in the rhizosphere microbiome composition by establishing beneficial microbial taxa and reducing the colonization by pathogens. These changes contributed to enhanced plant growth and disease suppression. Application of *Trichoderma harzianum* for biocontrol against *F. graminearum* causing *Fusarium* stalk rot on maize also resulted in beneficial alterations in the rhizosphere microbiome composition, mainly by increasing the abundance of the plant growth-promoting Acidobacteria and inhibiting *Fusarium* (Saravanakumar et al., 2017). Similarly, *Streptomyces* biocontrol strains applied to wheat seeds modulated the rhizosphere and endosphere microbiome compositions, causing significant shifts in the dominant microbial taxa at different wheat developmental stages and reducing the abundance of *Paenibacillus*. These changes resulted in the reduction of root disease and the promotion of wheat growth and productivity (Araujo et al., 2019). Similarly, *Bacillus velezensis* and *Pseudomonas fluorescens* applied for the biocontrol of *R. solanacearum* resulted in a profound shift in rhizosphere microbial communities. The shift was mainly due to the enrichment of Actinobacteria (which potentially have biocontrol activity), *Arthrobacter* (a known PGBR for tomato), and *Gaiella*. The three are members of the core microbiome in suppressive soils. The beneficial recruitment and stimulation of such beneficial microbial taxa were suggested to contribute to priming plant defense and promote plant growth (Elsayed et al., 2020).

These studies have demonstrated that biocontrol agents can significantly alter the rhizosphere microbiome by promoting beneficial microbial taxa and suppressing detrimental taxa. Thus, the manipulation of the rhizosphere microbiome through bioinoculation may be an integral component of biocontrol mechanisms and should be considered during the selection and development of novel biocontrol agents. This reflects the expectation that the rhizosphere competence and positive influence of bioinoculants on the rhizosphere microbiome will enhance the applicability of biocontrol agents in field conditions, and promote their establishment and root colonization. The inconsistency of the results obtained from single-strain inoculations can likely be attributed to the over-simplification of plant–microbe associations that result from the introduction of a single microbial genotype (Tosi et al., 2020). The efficacy of the inoculation process is entirely dependent on the survival, establishment, and performance of the single strain, which may be potentially outcompeted by the pre-existing resident microbiome.

#### 3.3.2. Consortia-based and synthetic microbial community inoculation

In addition to the classical approach of using single inoculants, studies have suggested the use of multi-species or synthetic microbial community inoculation. The use of synthetic microbial communities is a promising approach to promoting plant growth, increasing disease resistance, and improving stress tolerance. However, it is a challenging and complicated approach (Ahkami et al., 2017). Combining two or more strains requires critical consideration of the ecological interactions among the strains that are used. The six basic motifs of microbial interactions are commensalism, competition, predation, no interaction, cooperation, and amensalism (Großkopf and Soyer, 2014). Of these, cooperative interaction among the community members is the major driver of the assembly and functionality (Mitri and Foster, 2013). Along with reducing competition and parasitism, several other environmental factors (pH, temperature, nutrient availability, and host plant exudates) should be considered, since they also influence synthetic microbial community growth, stability, and sustainability (Ahkami et al., 2017).

Based on the knowledge of the host's selective recruitment of its rhizosphere microbiome, Niu et al. (2017) utilized a host-mediated approach followed by selective culturing to characterize and construct a simplified synthetic bacterial community composed of seven strains. The roles of each strain in sustaining community integrity were assessed. One keystone species was identified and the inoculation of maize with the synthetic microbial community resulted in the suppression of *F. verticillioides*, the causal agent of seedling blight. In another study that combined the host selective effect with organic amendment-associated microbiota, Tsolakidou et al. (2019) characterized a collection of microbes derived from compost that was enriched in tomato rhizosphere and constructed synthetic microbial communities from cultured microbes and characterized effective antagonistic species. The treatment with the synthesized microbial communities resulted in the promotion of tomato growth. Results were variable concerning *Arabidopsis*, plant growth promotion, and disease suppression of tomato, indicating the complexity of the interactions and the difficulty to predict the influence of the microbial consortia on host fitness. Similarly, Santhanam et al. (2019) studied the biocontrol activity of a consortium of five native isolates and described the suppression of sudden wilt disease caused by the *Fusarium–Alternaria* disease complex in tobacco. The biocontrol activity of the constructed consortium was attributed to the complementary action of the biofilm formed by three bacterial strains. Biofilm production was greater with the consortium than with the individual strains, along with siderophore production by two strains and the antifungal surfactin produced by one strain. Another study investigated the rhizosphere microbiome of garlic plants. The *Pseudomonas* genus was selected as the main PGBR at different plant developmental stages and a constructed synthetic microbial community comprised of six *Pseudomonas* strains promoted plant growth (Zhuang et al., 2021). More recently, Kaur et al. (2022) reported the positive influence of seed dressing with a synthetic microbial community of four bacterial strains on the growth of cotton. The growth benefit involved the direct provision of nutrients and hormones and indirect modulation of the rhizosphere microbiome composition.

The foregoing findings suggest that combining various strains within microbial consortia can have a positive impact, leading to improved stability and efficacy in various biocontrol applications. The use of multi-strain microbial consortia results in a more extensive genomic and functional diversity, which enhances metabolic efficiency and increased the ability to adapt to the surrounding environment (Ben Said and Or, 2017). However, these studies were limited to cultured microorganisms and thus neglected the potential benefits of uncultivable microorganisms. Additionally, the applicability of these findings may be hindered by the complex interactions between the microbes, soil, plant, and environmental conditions.

#### 3.3.3. Rhizosphere microbiome transplantation

Rhizosphere microbiome transplantation is an attractive approach that has the potential to circumvent the limitations associated with the lack of adaptability of microbial inoculants in bioinoculation applications. In addition, the approach could permit the incorporation of uncultivable microbes that could represent up to 99% of the microbes and are often overlooked in other methods. Despite its recent surge in popularity, transplantation of microbial communities for improved productivity through associated microbes is not a novel concept. It was practiced more than a century ago after the discovery of the legume nodulation microbes (Fred et al., 1932). The pioneering discovery by Hellriegel in 1886 regarding the nitrogen nutrition of leguminous plants was swiftly followed by the research of Salfeld in 1888. The latter is considered the father of soil-transfer inoculation, due to his groundbreaking field experiments on the distribution of soil to enhance the growth of leguminous crops (Otis, 1897). At that time, and long before the rise of the microbiome concept, bacteria-laden soil obtained from a field where a previous leguminous crop had produced nodules was incorporated into another field to encourage the nodulation of the new crops and improve productivity (Fred et al., 1932).

Increasing knowledge of the potential benefits of the rhizosphere microbiome on plant growth promotion and stress tolerance has spurred efforts to harness these benefits through the application of rhizo-microbiome transplantation techniques. Zolla et al. (2013) sampled the complete microbiomes of soils from different plant species (*Arabidopsis*, pine, and corn) and evaluated their potential to support the growth of *Arabidopsis* plants under drought stress conditions in whole microbiome transplantation experiments. The microbiome adapted to *Arabidopsis* improved drought tolerance in the target plants, as demonstrated by the increased plant biomass and decreased expression of drought response genes in *Arabidopsis*. Furthermore, microbiome composition analysis revealed that several bacterial genera with plant growth-promoting properties, including *Burkholderia, Phormidium, Bacillus*, and *Aminobacter*, were enriched in the *Arabidopsis*-adapted microbiome compared to the other microbiomes examined. These findings suggest the utility of whole rhizo-microbiome transplantation in supporting plant tolerance to environmental stress and highlight the importance of ensuring compatibility between the transplanted microbiome and the host plant.

The utilization of microbiome transplantation to enhance plant survival in highly petroleum-contaminated soils was investigated by Yergeau et al. (2015). Willow plants were grown on soil that had been irradiated to disrupt the native soil microbiome and reduce the microbial load. Subsequently, the plants were inoculated with various microbiomes sourced from the rhizosphere of willow plants that had displayed either optimal or suboptimal growth in petroleum-contaminated soils, as well as microbiomes sourced from bulk soils. Plants grown on soils with disrupted microbiomes exhibited reduced growth compared to plants treated with other inoculum treatments, which displayed increased biomass. The rhizosphere microbiomes displayed variations in composition immediately following treatment, but their composition tended to become more similar after 100 days of the plantation, likely as a result of the strong selective pressure exerted by the willow rhizosphere. These findings underscore the importance of considering several key factors when attempting to modulate the rhizosphere microbiome. These factors include the developmental stage of the host plant, the selective pressure exerted by the host plant on the microbiome assembly, the presence of keystone species within the community, the richness and diversity of the inoculum microbiome, and environmental conditions under which the plants are grown.

The potential of rhizo-microbiome transplantation to modulate plant physiological traits has also been investigated. Panke-Buisse et al. (2015) evaluated the ability of microbiome transplantation to influence flowering time. The authors described that microbiomes collected from the 10th generation of plantings that exhibited either early or late flowering were able to reproduce their effect on flowering time in the recipient plants. Analysis of microbiome composition revealed microbial variations that differentially influenced flowering time. Additionally, plants inoculated with microbiomes that induced late flowering displayed increased biomass, which was correlated with enhanced microbial extracellular enzyme activities associated with nitrogen mineralization in soils.

Jiang et al. (2022) recently established a systematic protocol for rhizosphere microbiome transplantation. The ability of the microbiome to enhance plant defense against soil-borne disease was used as an indicator of the success of the process. In this study, rhizosphere microbiomes collected from resistant plants were transplanted into susceptible plants. Successful microbiome transplantation was determined based on the suppression of bacterial wilt disease. Microbial profiling was performed to assess the ability of the donor rhizosphere microbiome to colonize the recipient plant rhizosphere. Culture-based *in vitro* assays were performed to explore the potential microbial taxa with suppressive abilities against *Ralstonia solanacearum*. The authors reported that only one of the six microbiomes used in the study was successful in rhizosphere microbiome transplantation. The suggested potential causes of unsuccessful transplantation included environmental, soil-related, and microbiome complexity-related factors. The findings of this study are a seminal contribution to the field, representing a shift from a trial-and-error-based approach to a more informed process for rhizosphere microbiome transplantation by characterizing the features that are necessary for successful transplantation (Jousset and Lee, 2023). This will pave the way for future applications of rhizosphere microbiome transplantation.

The phenomenon of transferring the entire microbiome, commonly referred to as microbiome transplantation, has been extensively studied in human microbiome research. The many purported benefits include, but are not limited to, the suppression of disease, modulation of immunity, and even reduction of obesity (Lee et al., 2019; Ser et al., 2021). In particular, the use of fecal microbiome transplantation in the treatment of *Clostridium difficile* infection has a success rate of over 90% (Bakken et al., 2011). Similarly, in the realm of plant science, the similarity of the rhizosphere microbiome to the human gut microbiome has been highlighted by the link of the rhizosphere microbiome to plant growth promotion, induction of plant resistance, and suppression of disease (Compant et al., 2010). Despite this, the applications of rhizosphere microbiome transplantation in plants have been relatively limited. This could be attributed to the complexity of the various variants and factors present in the plant rhizosphere. To address this limitation, optimization of factors, such as the compatibility of donor rhizo-microbiome to host plants, resident soil and rhizosphere microbiota in recipient plants, and the effect of the environment, could increase the scope of applications and maximize the benefits of the process.

**Figure 6:**
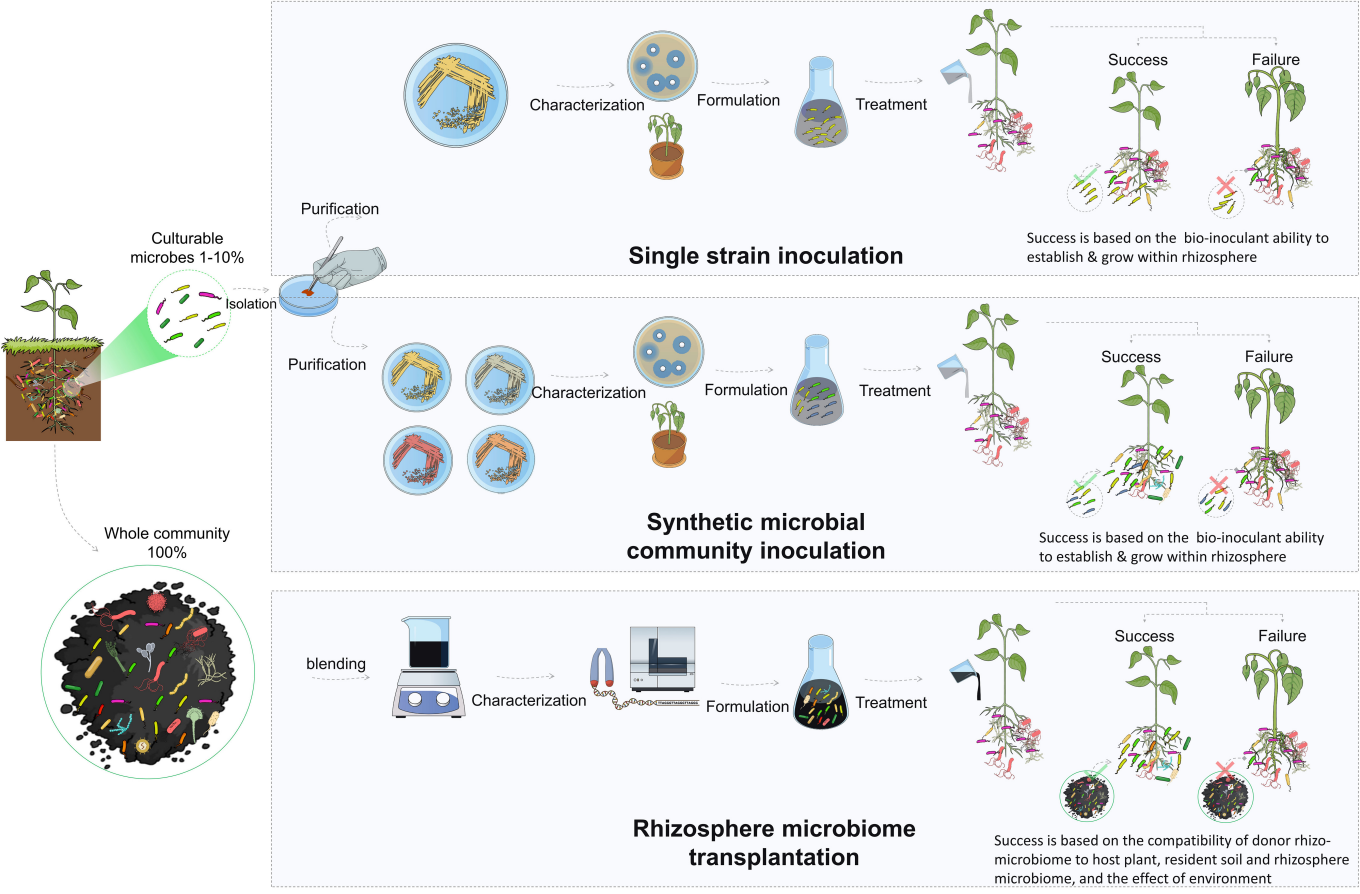
Illustration of the microbe-mediated rhizosphere microbiome engineering approach, starting from single-strain inoculation. This method involves the isolation, characterization, and application of a single strain. An alternative approach is the use of mixed consortia or synthetic microbial community inoculation, where two or more isolated and characterized strains are utilized for bioinoculation. These culture-based methods have limitations, as most microbes in the soil and rhizosphere are uncultivable. The success of the application largely depends on the ability of the bioinoculant to grow and establish its population within the rhizosphere microbiome community and ecosystem. In contrast, the rhizosphere microbiome transplantation represents a culture-independent approach, where the entire community is transferred from the donor to the recipient plant. This approach includes the whole community of culturable and unculturable microbes and eliminates limitations associated with the lack of adaptability of microbial inoculants in culture-based methods.

The microbe-based approaches for rhizosphere microbiome engineering are summarized in Figure 6.

## 4. Concluding remarks and future perspectives

The rhizosphere is a critical element for plant growth and health. The vital role of the rhizosphere microbiome in supporting plant growth and tolerance to stress has been experimentally confirmed. Recent advances in the metagenomic analysis have greatly improved our understanding of the diversity and complexity of microorganisms present in the rhizosphere, as well as their interactions with plants and other microorganisms. To maximize the benefits of rhizosphere-based methods, a thorough understanding of the factors that govern the assembly and functional capacity of the rhizosphere microbiome is necessary. These factors can be broadly categorized as host plant-related, soil-related, and microbe-related determinants.

In addition to understanding the rhizosphere microbiome assembly, methods have been proposed to manipulate and engineer the microbiome to promote plant growth, adaptation, and survival under harsh conditions, or to enhance the tolerance to stress and disease. These methods can also be broadly categorized as host plant-related, soil-related, and microbe-related approaches. The rhizosphere microbiome engineering methods have shown considerable success and hold promise for improving the quality and productivity of agricultural crops. However, much remains to be understood about the specific mechanisms by which microorganisms interact with plants, and how these interactions can be harnessed to optimize plant growth and health. Further research is required to fully comprehend the complex interactions within the rhizosphere and to develop effective strategies for microbiome engineering. Additionally, the integration of sustainable and environmentally friendly agricultural practices that consider the rhizosphere microbiome can play a crucial role in achieving a more sustainable future.

We believe that integrating the modulation of the different variants involved in the assembly and functionality of the rhizosphere microbiome, including host plant, soil-related factors, and resident microbiome and microbe–microbe interactions, will be crucial to improve the success rate of rhizosphere microbiome engineering and enhance the benefits to plants. Future research discoveries will help fully realize the potential of rhizosphere microbiome engineering in plants.

## Author contributions

MM and Y-SS conceived and designed the concept. IP and MM collected and performed literature data interpretation. IP, MM, and Y-SS wrote and revised the manuscript. All authors read and approved the manuscript.
